# On the Relation Between Cross-Linguistic Influence, Between-Language Priming and Language Proficiency: Priming of Ungrammatical Adjective Placement in Bilingual Spanish-Dutch and French-Dutch Children

**DOI:** 10.1162/opmi_a_00105

**Published:** 2023-09-20

**Authors:** Chantal van Dijk, Sharon Unsworth

**Affiliations:** Centre for Language Studies, Radboud University, Nijmegen, The Netherlands; English and American Studies, Technische Universität Braunschweig, Braunschweig, Germany; Institute of Linguistics, Universität Stuttgart, Stuttgart, Germany

**Keywords:** structural priming, shared syntax, proficiency, cross-linguistic influence, ungrammatical structures, bilingual children

## Abstract

After hearing a structure in one language, bilinguals are more likely to produce the same structure in their other language. Such between-language priming is often interpreted as evidence for shared syntactic representations between a bilingual’s two languages and is positively related to proficiency. Recently, shared syntactic structures and structural priming have been invoked to explain cross-linguistic influence in bilingual children. This paper examines the relation between cross-linguistic influence, between-language priming and language proficiency. Almost all studies on between-language priming have focussed on grammatical structures. However, cross-linguistic influence has also been found to result in *ungrammatical* structures. In this study, we investigated whether ungrammatical adjective placement can be primed from a Germanic language to a Romance language and vice versa, and how to best account for any such priming. Furthermore, we examined the role of proficiency in explaining priming effects and whether this fits with an error-based learning account. Our results show that it is possible to prime ungrammatical structures, that this is lexically constrained, and that it is more likely to occur at lower levels of proficiency. We argue that the same mechanisms underlying grammatical priming can also explain our findings of ungrammatical priming.

## INTRODUCTION

Bilingual adults are often thought to develop shared syntactic representations between their languages (e.g., Hartsuiker & Bernolet, 2017; Hartsuiker et al., 2004). Evidence for shared syntax comes from between-language structural priming (e.g., Hartsuiker et al., 2004; Loebell & Bock, 2003), that is, the observation that a structure in one language can prime subsequent use of the same structure in the other language. Further evidence for shared syntax in bilingual adults comes from speech production data (Runnqvist et al., 2013) showing that the speed with which bilingual adults produced a structure in one language was modulated not only by its structural similarity to the other language, but also by the frequency of said structure in both languages. Proficiency has also been found to modulate between-language priming effects in bilingual adults (Hartsuiker & Bernolet, 2017).

Shared syntactic structures and structural priming have furthermore been invoked to explain one of the most intensively studied topics in bilingualism, cross-linguistic influence (e.g., Hervé et al., 2016; Loebell & Bock, 2003). Cross-linguistic influence involves the overuse or overacceptance of a particular (morpho)syntactic property in one of a bilingual’s languages under influence of the other (Serratrice, 2016; van Dijk et al., 2022), and crucially, occurs not only in bilingual but also in monolingual contexts. Frequent exposure to a particular—shared—morphosyntactic property in one language primes the use of this property in the other language (e.g., Serratrice, 2007, 2016). Hence, through priming of shared structures, exposure to one language over time modulates the frequency with which similar structures in the other language are used.

Evidence for between-language structural priming overwhelmingly comes from structures that are grammatical in the prime and target language. Cross-linguistic influence has however been observed to result in ungrammatical utterances as well. For example, Romance-Germanic bilingual children have been found to produce ungrammatical postnominal N(oun)-Adj(ective) orders in their Germanic language (e.g., *an apple green) under influence of their Romance language, as well as ungrammatical prenominal Adj-N orders in their Romance language (e.g., *une verte pomme, an apple green) under influence of their Germanic language (Granfeldt, 2000; Nicoladis, 2006). We investigate whether such ungrammatical cross-linguistic influence is also the result of between-language priming. In doing so, we explore the mechanisms underpinning structural priming and cross-linguistic influence more generally. Furthermore, we ask whether proficiency modulates any between-language priming resulting in ungrammatical structures.

### Between-Language Priming, Shared Syntax and the Role of Proficiency

Structural priming is typically accounted for by two mechanisms: residual activation (e.g., Pickering & Branigan, 1998) and implicit learning (e.g., Chang et al., 2006). On the residual co-activation account, priming is lexically driven (e.g., Levelt et al., 1999; Pickering & Branigan, 1998). Lemmas (e.g., the lemma for *green*) are connected to conceptual (e.g., the concept *green*) and combinatorial nodes (e.g., an Adj-N node specifying the prenominal adjective order). When a listener hears a sentence, activation will spread to the relevant lemmas, their conceptual nodes and any relevant combinatorial node(s). Recently activated combinatorial nodes are left with some residual activation, making it easier to re-activate them for subsequent use. Because residual activation decays over time, any priming effects should be short-lived.

On an implicit learning account (e.g., Chang et al., 2000, 2006; see also Jaeger & Snider, 2013; Reitter et al., 2011), structural priming is the result of surprisal and error. Listeners predict the structure of an upcoming sentence on a word-by-word basis, and when the structure they predict is not the one they hear, that is, when they are surprised, an error signal is produced. In an error-based learning account of priming, such as the dual-path model (e.g., Chang et al., 2000, 2006), this error results in the connection weights associated with the structural representation in question being updated, making the language system more likely to subsequently predict and produce that same structure. Crucially, on an error-based learning account, and in contrast with the residual-activation account, weight changes accumulate over time and result in learning. Priming effects are thus long-lasting.

**Figure 1.**
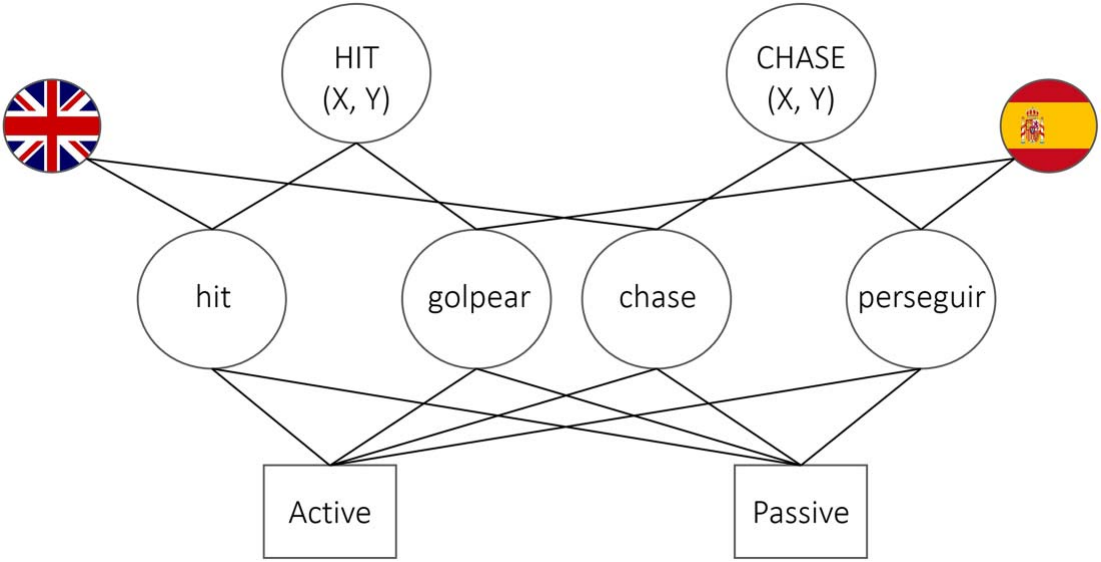
Example of the shared-syntax (and shared-lexicon) model adapted from Hartsuiker et al. (2004). The conceptual nodes HIT and CHASE are connected to their English lemmas *hit* and *chase* and their Spanish lemmas *golpear* and *perseguir*. Language membership is indicated by the connections to language nodes. All lemmas are connected to the ‘active’ and ‘passive’ combinatorial nodes. Note that lemmas in Hartsuiker et al. (2004) are also connected to a categorical ‘verb’ node, excluded here for sake of simplicity.

Assuming that syntactic representations which are similar enough are indeed shared (Hartsuiker et al., 2004), both the residual activation and error-based learning accounts can explain structural priming between languages. On a residual activation account, exposure to a structure in one language primes the shared structural representation for subsequent use. For example, in Figure 1 the English and Spanish verbs “hit” and “chase” are connected to the same combinatorial nodes. Consequently, when a Spanish-English bilingual is primed with a passive structure in English, she will be more likely to reactivate the passive node for subsequent use, either in English or in Spanish.

The learning mechanism underlying error-based learning accounts has also been argued to be language-independent (Janciauskas & Chang, 2018). Recent evidence from computational modelling suggests that this proposal is correct (e.g., Hwang & Shin, 2019; Khoe et al., 2023; Tsoukala et al., 2017). As such, when a particular structure is frequent in one language, the same structure is also predicted and produced more frequently in the other language (e.g., Hervé et al., 2016; Khoe et al., 2020, 2023). Error-based learning furthermore provides an explanation for the frequently-made observation in bilingual children’s productions that cross-linguistic influence takes place in monolingual contexts (Serratrice, 2013; van Dijk et al., 2022). More specifically, exposure over time to (frequent) structures in one language results in long-term adaptation to that structure. If adaptation is language-independent, bilingual children are likely to use the same structure more often in *both* of their languages (see Runnqvist et al., 2013 for a similar approach with adults).

On both accounts—error-based learning and residual activation—between-language priming develops as a function of proficiency. According to Hartsuiker and Bernolet’s (2017) developmental model of shared syntax, learners develop abstract structural representations and share these between languages only when they are sufficiently proficient in their L2. Consequently, the developmental model predicts a positive relationship between L2 proficiency and priming as abstract shared structures are in the process of being acquired. Similarly, a minimal level of proficiency is needed for error-based learning to take place (e.g., Chang et al., 2012; Janciauskas & Chang, 2018): a linguistic structure must have first been encountered and to some extent stored before it can be predicted, and its connection weights adapted. From the moment abstract representations are acquired, an error-based learning account predicts a *negative* relation between proficiency and the magnitude of any priming effects. In the early stages of acquisition, children’s representations are less stable and therefore more malleable, which means that prediction errors are expected to be stronger and lead to greater learning and priming effects (e.g., Fazekas et al., 2020; Jaeger & Snider, 2008; Peter et al., 2015). Assuming age is a reasonable proxy for proficiency, studies on computational modelling (e.g., Chang et al., 2006) and priming in monolingual children (e.g., Rowland et al., 2012) suggest that this is the case.

To summarise, the two main accounts of structural priming—residual activation and error-based learning—can both straightforwardly explain between-language priming effects in bilingual adults. Both accounts rely on some level of sharing between structural representations in a bilingual’s two languages, and structural similarity is thought to be necessary for sharing to take place. Typically, such similarity involves what in the developmental literature has been referred to as *partial* overlap between the two languages (e.g., the prenominal Adj-N order being present in both English and French, but the postnominal N-Adj order existing in French only) and *complete* overlap (e.g., the prepositional object and double object dative structure existing in both Dutch and English; Unsworth, 2013; van Dijk et al., 2022). Cross-linguistic influence in bilingual children can however result in ungrammaticality. It remains unclear whether between-language priming is also the mechanism underlying such ungrammatical cross-linguistic influence and, if so, whether and how it is related to language proficiency.

### Between-Language Priming, Ungrammatical Structures and Cross-Linguistic Influence

There is some evidence that structures with a similar function but a different word order can prime each other between languages (e.g., Chen et al., 2013; Hwang & Shin, 2019; Khoe et al., 2023; Shin & Christianson, 2009). For example, Hwang and Shin (2019) found that Mandarin passive structures primed English passive structures in Mandarin-English bilingual adults even though these structures differ in word order (i.e., SOV in Mandarin and SVO in English). However, in other studies priming has not been found without exact word order overlap (e.g., Bernolet et al., 2007; Loebell & Bock, 2003).

Studies aiming to prime ungrammatical word orders are rare. One exception is Hsin et al. (2013). In this study, Spanish-English bilingual children described pictures in Spanish using adjective-noun combinations. The default grammatical position for adjectives in Spanish is postnominal (e.g., *libro_N_ abierto_ADJ_*, “open book”).^1^ Children were more likely to produce incorrect Spanish prenominal orders (e.g., *abierto_ADJ_ libro_N_) after being primed with prenominal Adj-N orders in English compared to a neutral baseline. In other words, exposure to a structure in one language primed the same but ungrammatical structure in the other. Hsin et al. argued that their results were evidence for broad sharing of (un)grammatical structures between languages, although they did not specify what exactly is shared.

**Figure 2.**
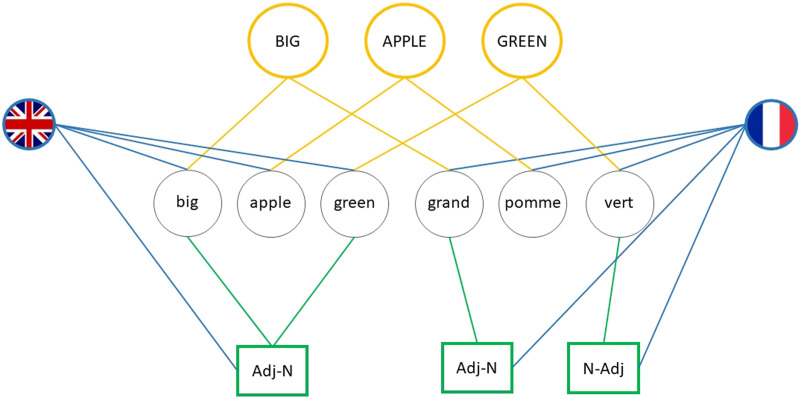
Schematic representation of the English lemmas *big*, *green*, and *apple* and the French lemmas *grand*, *vert*, and *pomme* and their connections to their conceptual (in yellow), structural (in green) and language nodes (in blue). Based on Nicoladis (2006).

Nicoladis (2006) proposed an alternative explanation for why bilingual Romance-Germanic children sometimes produce ungrammatical N-Adj structures in their Germanic language (see also Kupisch, 2014). The principles underlying her account (based on the speech production models in Costa, 2004 and Ferreira & Dell, 2000) are similar to those of the residual activation account. However, crucially, the combinatorial nodes (or ‘syntactic frames’) are not shared across languages (Figure 2; see Kantola and van Gompel (2011) for a similar approach). According to Nicoladis, a child’s intention to produce an adjective-noun structure (e.g., *green apple*) activates the corresponding conceptual nodes for GREEN and APPLE, shared between languages. In turn, activation spreads from these conceptual nodes to the English lemmas for *green* and *apple* and subsequently to the prenominal Adj-N node. However, because GREEN is also connected to the French lemma *vert*, which is also activated, activation is also passed on to the postnominal N-Adj node. Consequently, the prenominal and postnominal nodes compete for selection. Because the child intends to produce an English sentence, she is more likely to select the correct prenominal node. However, in some cases, activation of the postnominal N-Adj node might be strong enough to result in an ungrammatical postnominal N-Adj order with English lemmas (i.e., *apple green). In other words, on this account, cross-linguistic influence is considered an epiphenomenon of speech production (Nicoladis, 2006, p. 26).

On Nicoladis’ (2006) account, between-language priming of ungrammatical adjective orders is only expected for adjectives which appear in different positions in the two languages. More specifically, only English adjectives whose translation equivalent in French is postnominal (e.g., *green* but not *big*) will activate the French postnominal N-Adj node. The extent of any such activation may depend on recent (on a residual activation account) and/or frequent (on an error-based learning account) exposure to this structure in French. When the French translation-equivalent of an English adjective is prenominal, the corresponding French lemma will only activate the prenominal Adj-N node. Consequently, the N-Adj order will not be considered for selection, regardless of whether it has been used previously (i.e., as part of a French prime).

It is an open question whether and how proficiency relates to priming of ungrammatical structures. The developmental account of shared syntax assumes that priming will increase with proficiency (e.g., Hartsuiker & Bernolet, 2017) for structures which are structurally similar. Given that this is not the case for structures which are ungrammatical in one of the two languages, no such relation between proficiency and priming is expected. On Nicoladis’ account, priming leading to ungrammaticality results from the erroneous selection of a structure available in the ‘other’ language, due to (co-)activation of the conceptual and language nodes. Bilinguals may be more prone to making such errors when their syntactic representations are weaker, which would be in line with error-based learning accounts (e.g., Fazekas et al., 2020; Jaeger & Snider, 2008; Peter et al., 2015). The weaker a syntactic structure, the larger the surprisal effect when encountering such structure, and hence the stronger the change in weights of the associated connections, resulting in a larger priming effect. Indeed, Nicoladis (2006) observed a negative relation between the number of English adjective-noun reversals in French-English bilingual children and children’s English proficiency, as well as between the number of French adjective-noun reversals and children’s French proficiency, suggesting that ungrammatical between-language priming might be stronger with lower proficiency.

### The Present Study

In this study we investigate (i) whether ungrammatical adjective placement can be primed from a Germanic language to a Romance language and vice versa; (ii) the extent to which such priming is lexically constrained, and (iii) the role of proficiency in explaining priming effects. The study consists of two experiments. Experiment 1 tested whether the ungrammatical postnominal N-Adj order in a Germanic language, Dutch, can be primed by the same (grammatical) order in a Romance language, Spanish. We predicted that if between-language priming is the mechanism behind cross-linguistic influence (e.g., Hervé et al., 2016; Serratrice, 2016), postnominal N-Adj orders in Spanish should prime ungrammatical postnominal N-Adj orders in Dutch.

Experiment 2 investigated the extent to which any between-language priming of ungrammatical structures is lexically constrained. More specifically, we tested whether between-language priming of ungrammatical adjective-noun combinations in French and Dutch is dependent on the adjectives produced. Adjective placement was primed in both directions. We hypothesized that if the postnominal N-Adj node is connected to lemmas in French only, a postnominal prime in French should prime the postnominal order with Dutch adjectives whose French translation equivalent occurs in postnominal position (e.g., *green*), but not with those whose French translation equivalent occurs in prenominal position (e.g., *big*). For Dutch-to-French priming, we expected priming of the prenominal Adj-N order to occur irrespective of the type of adjective, because all Dutch adjectives are prenominal.

Finally, in both experiments, we investigated effects of language proficiency on between-language priming. We hypothesised that if between-language priming results from error-based learning, stronger priming effects are expected with lower proficiency. Our prediction concerns proficiency in *both* languages. This is because—in contrast to adult (or sequential) bilinguals—in child (or simultaneous) bilinguals, both languages are developing at the same time.

## EXPERIMENT 1: SPANISH-TO-DUTCH PRIMING

### Method

#### Participants.

Twenty-nine Spanish-Dutch bilingual children between 5 and 8 years old (*M* = 6.3; *SD* = 0.8) participated. All were exposed to both Spanish and Dutch from birth, with one exception (age of first exposure to Dutch at 1;6). This child’s behaviour did not stand out in any of the analyses (based on Cook’s distance). All children were living in the Netherlands at the time of testing in two-parent households. Three quarters of the Spanish-Dutch bilingual children (*N* = 22) had one parent who (mostly) spoke Dutch to them and the other (mostly) Spanish. In three families, one parent (mostly) used Dutch and the other parent both languages, in another three families both parents (mostly) spoke Spanish and children were exposed to Dutch outside the home (i.e., at daycare and then at school), and in the remaining family both parents mostly spoke Dutch at home and the child heard Spanish from the au-pair.

#### Vocabulary Task.

Children’s language proficiency was tested using a picture-naming task, the Cross-linguistic Lexical Task (CLT; Haman et al., 2015; van Wonderen & Unsworth, 2021), which was designed to be comparable across languages by using the same two language-specific properties to select target words (i.e., the target words’ age of acquisition and complexity). The task included 30 nouns and 30 verbs, presented separately, and counterbalanced across children. Accuracy scores were calculated. Overall, children scored higher in Dutch than in Spanish (*t* = 3.9; *df* = 45.9; *p* < .001; see Table 1), and scores in the two languages were not correlated (Pearson’s *r* = .076; *p* = .700).

**Table 1.** Experiment 1, Spanish-Dutch bilingual children: Children’s mean scores, standard deviations (in brackets) and range on the vocabulary and digit span tasks.

**Productive vocabulary**	**Dutch**	**Spanish**
(Proportion correct)	0.72 (0.13)	0.54 (0.21)
	0.44–0.94	0.10–0.85
**Digit span** ^**a**^	**Forward**	**Backward**
(Raw scores)	21.4 (3.5)	7.6 (3.7)
	12–30	0–16

^a^ Data from one child is missing.

#### Short-Term and Working Memory Task.

Previous research (Foltz et al., 2015) with monolingual children has found that the magnitude of priming effects may be related to their short-term and working memory capacity. For this reason, we included a Dutch digit span task from the Automated Working Memory Assessment (AWMA; Alloway, 2012) in our test battery (Table 1). Children received one point for each correctly repeated/reversed digit sequence. Sequences ranged from 1 to 8 in the forward block (max. 48 points; proxy of short-term memory capacity) and from 2 to 7 in the backward block (max. 36 points; proxy of working memory capacity).

#### Priming Task.

The structural priming task was designed to elicit noun plus adjective combinations and following Messenger et al. (2011), consisted of a card game (“Snap”). Five colour adjectives were chosen in Dutch and Spanish (i.e., *rood-rojo* ‘red’, *blauw-azul* ‘blue’, *geel-amarillo* ‘yellow’, *oranje-naranja* ‘orange’, and *groen-verde* ‘green’). These colours were paired with eight different characters (i.e., *astronaut-astronauta* ‘astronaut’, *boer-agriculturo* ‘farmer’, *brandweerman-bombero* ‘firefighter’*, dokter-médico* ‘doctor’, *piloot-piloto* ‘pilot’, *piraat-pirata* ‘pirate’ *schilder-pintor* ‘painter’, *tandarts-dentista* ‘dentist’). We started by creating all possible adjective-noun combinations. Subsequently, we made a random selection from this pool to arrive at 16 critical pairs (i.e., same character, but different colour) plus eight identical pairs (e.g., a red doctor)—not included in the analyses—used to implement the game element of the task. We made sure to include approximately the same number of items per colour. These were subsequently included in four experimental lists and the order of experimental items and fillers (containing possessive structures where prime and target pictures differed, used for another study; Unsworth, in press) was pseudo-randomized such that there were at least two intervening fillers between each item.

Children were told they were going to play a game with cards. As part of the game, the experimenter and the child would take turns to draw a card from a (pre-ordered) pile next to them and describe what they saw and place the card in the middle of the table. Children were informed that the experimenter always went first and that sometimes the two cards were different and sometimes they were the same. When they were the same, both experimenter and child had to place their hands on top of the cards as quickly as possible and shout “Hetzelfde!” (‘Same’). The person who was first to do so was allowed to keep the cards and the person with the most cards at the end was the winner. This was always the child. Children completed the priming task twice, once with Dutch primes and Dutch targets (i.e., within-language priming), and once with Spanish primes and Dutch targets (i.e., between-language priming). In the Dutch-to-Dutch task, the primes were always prenominal Adj-N orders whereas in the Spanish-to-Dutch task, the primes were postnominal N-Adj orders. The Dutch-to-Dutch task served as a baseline.

#### Procedure.

Children were tested in two separate sessions, which typically were scheduled with one week in between. The tester was a native speaker of Dutch and had near-native proficiency in Spanish. In the within-language session (always first), the tester and child always spoke Dutch. This session was completely monolingual, and the order of tasks was: (1) first half of the priming task; (2) Dutch vocabulary task; (3) second half of the priming task; (4) Dutch sentence repetition task (as part of a separate project); and (5) digit span task. In the between-language session the tester always spoke Spanish and the child spoke Dutch during the priming task. This session was bilingual and the order of tasks was: (1) first half of the priming task; (2) Spanish vocabulary task; (3) second half of the priming task; (4) Spanish sentence repetition task (as part of a separate project). Each session lasted approximately one hour.

Parents gave informed consent. Children were tested individually in a quiet room at home or school by a native or near-native research assistant. To remind the child which language to speak, two small flags were introduced at the start of the bilingual session (a Dutch one and a Spanish one); the relevant flag was placed in front of the child and changed accordingly as the test session progressed. Children’s responses were recorded using an audio recorder and where necessary checked afterwards.

#### Coding and Analyses.

Answers on the priming tasks were coded as *prenominal* when children produced adjective-noun combinations with the adjective preceding the noun (e.g., *rode hond*, “red dog”) and *postnominal* when the adjective followed the noun. Responses without a noun or adjective or containing predicative uses of the adjective (e.g., *de hond is rood*, “the dog is red”) were coded as *other* and subsequently excluded from the analyses (3.8% (30/792) in the between-language session; 0 in the within-language session; see S1 in the supplementary materials for a breakdown of the *other* responses). The proportion of postnominal N-Adj responses was calculated by dividing the number of postnominal responses by the total of postnominal plus prenominal responses.

We analyzed the proportion of postnominal responses using generalized linear mixed models from the lme4 package in R (version 1.1-28; Bates et al., 2015). All scripts and model output can be found on OSF. An effect was deemed significant when its *z*-value had a *p*-value below 0.05 (lmerTest package, version 3.1-3; Kuznetsova et al., 2017). All models contained random intercepts by *child* and, if this did not result in convergence errors, *item*.^2^ In a first step, we created a base model, which included all background variables: *trial number*, *age*, *forward digit span*, and *backward digit span*. Non-significant background variables were dropped from the model before proceeding to the next step (see OSF for our decision process). In a second step, we investigated proficiency effects by adding Dutch and Spanish vocabulary scores as fixed effects. All fixed effects were centred around their grand mean.

We inspected the assumptions and fit of all models by visually inspecting the distribution of the observed, expected and standardized residuals and by performing nonparametric dispersion tests (DHARMa package, version 0.4.5; Hartig, 2022), by calculating the models’ conditional and marginal *r*^2^ (performance package, version 0.9.0, Lüdecke et al., 2021), and by calculating and inspecting Cook’s distance and DF Beta values for each child (influence package, version 0.9-9; Nieuwenhuis et al., 2012). Influential cases were children with a Cook’s distance larger than 4 divided by the number of children in the dataset (Van der Meer et al., 2010) and we re-ran models without these children. In case the removal of influential cases resulted in different model outcomes (whether an estimate was significant or not or changed direction) we report the summary of the model with and without the influential cases. Where removal of the influential cases did not result in different model outcomes, we report this model only. For summaries of models including all cases, we refer the reader to the OSF database.

### Results

In the within-language priming task (Dutch-to-Dutch), children always produced prenominal Adj-N orders (*N* = 232). In the between-language priming task (Spanish-to-Dutch), children produced 39 postnominal N-Adj orders (*M* = 0.21; *SD* = 0.28; range = 0–1) and 180 prenominal Adj-N orders.^3^

Because children never produced postnominal structures in the within-language session, we could not compare the two language sessions in a generalized linear mixed model. Instead, we tested whether children’s production of postnominal orders in the between-language session was significantly greater than 0, suggesting that children were primed. A one-tailed binomial test showed that this was the case (*p* < .001; 95% CI [0.14–1.0]).

#### Vocabulary.

Children’s Dutch vocabulary scores correlated negatively with the proportion of postnominal orders they produced (*r* = −.39; *p* = .037); the same tendency was evident for Spanish vocabulary, albeit not significant (*r* = −.29; *p* = .12). In a model with all children, Spanish vocabulary was a significant predictor of children’s priming behaviour (*B* = −5.17; *SE* = 2.11; *z* = −2.45; *p* = .014) and Dutch vocabulary approached significance (*B* = −6.74; *SE* = 3.69; *z* = −1.83; *p* = .068). One child was identified as influential case based on her Cook’s distance. In the model without this child (Table 2), Dutch and Spanish vocabulary were significant predictors: children were more likely to produce a postnominal adjective in Dutch, as their Dutch and Spanish vocabulary decreased. Furthermore, the likelihood of producing a postnominal adjective in Dutch significantly decreased as a function of trial number.

**Table 2.** Experiment 1, Spanish-Dutch bilingual children: Summary of generalized linear mixed effects model with the fixed effects of children’s Spanish and Dutch vocabulary scores (one influential case removed).

**Random effects**				
**Groups**	**Name**	**Variance**	***SD***	
Participant	(Intercept)	1.185	1.089	
Number of observations: 203	Participants: 27^a^			
**Fixed effects**				
	***B***	***SE***	***z***	***p***
(Intercept)	−2.31	0.45	−5.09	< .001
Vocabulary Dutch	−9.10	3.56	−2.56	.011
Vocabulary Spanish	−3.85	1.84	−2.09	.036
Digit span forward	−0.23	0.14	−1.69	.091
Trial number	−0.12	0.04	−2.95	.003

^a^ Data from one additional child was excluded because she did not complete the digit span task.

### Discussion

The results of Experiment 1 confirm our first hypothesis. The ungrammatical postnominal N-Adj order was primed cross-linguistically from Spanish into Dutch, extending Hsin et al.’s (2013) observation of priming of ungrammatical prenominal Adj-N orders from a Germanic into a Romance language to priming of N-Adj orders from a Romance into a Germanic language, and in line with studies on cross-linguistic influence where children produced ungrammatical postnominal N-Adj structures in Germanic languages under influence of their Romance language (e.g., Nicoladis, 2006). Hence, the results are consistent with the claim that between-language priming is the mechanism behind cross-linguistic influence (e.g., Serratrice, 2016), and that this can result in ungrammatical structures.

Our results also align with previous studies with bilingual adults showing that word order overlap between languages is not required for between-language priming to take place (e.g., Chen et al., 2013; Shin & Christianson 2009). Adopting a two-stage model of sentence production (Bock & Levelt, 1994), Chen et al. (2013) argued that between-language priming without word order overlap may result from shared representations before linear order relations (i.e., word order) are assigned (see also Ziegler & Snedeker, 2018). Our findings could be explained similarly, by assuming that all adjectives are generated in prenominal position in both Germanic and Romance languages and that the N-Adj word order is subsequently realised by moving the N to a higher syntactic position (e.g., Cinque, 2010). Further research would be needed to determine the extent to which this theoretical account of adjective placement corresponds to the two stages of language production invoked by Chen et al., and which representations would be shared exactly.

Also in line with previous studies on cross-linguistic influence is the observation that some but not all children showed priming effects. We hypothesised that some of the variation in children’s priming behaviour would be accounted for by differences in proficiency. The observed significant negative effects of children’s Dutch and Spanish vocabulary scores are consistent with an error-based learning account of priming. Additional evidence for error-based learning of ungrammatical priming comes from the observed negative effect of trial number on children’s production of postnominal adjectives, which suggests that prime surprisal—and, thus, the priming effect—decreased with increasing exposure to Spanish postnominal orders. Furthermore, a negative relation with priming for proficiency in both Spanish and Dutch suggests that the stability of structural representations in both the bilingual children’s languages matters for the priming of ungrammatical structures. We return to this issue in the General Discussion.

Experiment 1 showed that it is possible to prime ungrammatical adjective-noun combinations between languages, but it did not allow us to investigate whether such priming is lexically restricted. We answer this question in Experiment 2 by manipulating the placement of French adjectives. In addition, Experiment 1 leaves open whether the production of ungrammatical orders in Dutch was a direct consequence of the Spanish N-Adj primes or a result of children being in a bilingual language mode in the Spanish-Dutch priming block. That is, purely the fact that children were listening to Spanish in the Spanish-Dutch priming block might have strongly activated the Spanish N-Adj structure in general, resulting in children sometimes producing the Spanish postnominal N-Adj order in Dutch, irrespective of the primes. To address this possibility in Experiment 2, we included two priming blocks: a postnominal block with French N-Adj primes and a prenominal block with French Adj-N primes. We expected that if Romance N-Adj structures prime the same order in Dutch, the number of Dutch N-Adj orders should be higher in the postnominal than in the prenominal priming block. We furthermore examined bidirectional language priming to test whether the proficiency effects observed in Experiment 1 hold in both directions. Finally, we measured children’s baseline production of adjective-noun combinations in Dutch and French in the absence of any priming to establish whether bilingual children showed evidence of cross-linguistic influence before being primed.

## EXPERIMENT 2: FRENCH-TO-DUTCH AND DUTCH-TO-FRENCH PRIMING

### Method

#### Participants.

Thirty French-Dutch bilingual children between 4 and 8 years old (*M* = 6.4; *SD* = 1.3) participated. All children acquired French and Dutch from birth, with one exception (age of first exposed to Dutch at 1;3). This child did not stand out in any of the analyses (based on Cook’s distance). All children were living in the Netherlands at the time of testing in two-parent households. Most (*N* = 23) had one parent who (mostly) spoke Dutch to them and the other (mostly) French. There were four families where one parent spoke Dutch and the other both languages, one family where both parents spoke both languages, and one family where both parents spoke French and the child was exposed to Dutch outside the home (i.e., at daycare and then at school). This information was missing for one child.

#### Vocabulary.

Children’s receptive vocabulary scores were measured using the Peabody Picture Vocabulary Task in Dutch (PPVT-III-NL; Dunn et al., 2005) and in French (Échelle de Vocabulaire en Images Peabody; Dunn et al., 1993). Both tasks required children to match a word they heard with one out of four pictures. On average, children scored higher on Dutch than French (Table 3; *t* = 4.8; *df* = 4.8; *p* < .001). There was a significant and positive strong correlation between children’s Dutch and French scores (raw scores: *r* = .56; *p* = .003; standardized scores: *r* = .53; *p* = .005).

**Table 3.** Experiment 2, French-Dutch bilingual children: Children’s mean scores, standard deviations (in brackets) and range on the vocabulary and digit span tasks.

**Receptive vocabulary**	**Dutch** ^**a**^	**French** ^**b**^
Raw scores	92.3 (14.7); 68–121	49.2 (23.1); 14–96
Standardized scores	107.0 (9.8); 89–127	89.1 (17.4); 63–122
**Digit span** ^**c**^	**Forward**	**Backward**
Raw scores	21.9 (4.2); 13–30	8.0 (2.9); 1–13

^a^ Data from 3 children are missing.

^b^ Data from 1 child are missing.

^c^ Data from 4 children are missing.

#### Short-Term and Working Memory.

See Experiment 1. Results are shown in Table 3.

#### Priming Task.

The structural priming task followed the same design as Experiment 1. First, twelve nouns (i.e., *pompier*/*brandweerman* ‘firefighter’*, médecin*/*dokter* ‘doctor’, *homme*/*man* ‘man’, *garçon*/*jongen* ‘boy’, *roi*/*koning* ‘king’, *singe*/*aap* ‘monkey’, *ours*/*beer* ‘bear’, *coq*/*haan* ‘cockerel’, *chien*/*hond* ‘dog’, *chat*/*kat* ‘cat’, *canard*/*eend* ‘duck’, and *éléphant*/*olifant* ‘elephant’) and twelve adjectives were selected based on their imageability and age of acquisition. Six adjectives were prenominal in French (i.e., *gros*/*dik* ‘fat’, *petit*/*klein* ‘small’, *beau*/*mooi* ‘beautiful’, *jeune*/*jong* ‘young’, *grand*/*groot* ‘big’, and *vieux*/*oud* ‘old’) and the other six postnominal (i.e., *jaune*/*geel* ‘yellow’, *vert*/*groen* ‘green’, *rouge*/*rood* ‘red’, *méchant*/*boos* ‘angry’, *trieste*/*verdrietig* ‘sad’, and *heureux*/*blij* ‘happy’).^4^

Out of all possible 144 adjective-noun combinations we selected 48 prime, 48 target and 24 baseline items (see below). Each adjective appeared four times as prime, four times as target and two times as baseline item. Prime items were combined with four different target items: two prenominal and two postnominal (or for Dutch, items whose translation equivalent in French was postnominal). Hence, target items appeared with four different prime items. There was no lexical overlap between items in a prime-target pair. The game element was implemented using twenty-two filler items which used NP-PP structures (e.g., *un bébé avec des jouets* / *een baby met speelgoed* ‘a baby with toys’), some (*n* = 10) of which were identical.

Prime-target combinations were pseudo-randomly distributed with the fillers over four lists such that (1) each adjective appeared four times as prime and four times as target in a list; (2) each prime/target adjective was combined two times with a prenominal target/prime and two times with a postnominal target/prime adjective; and (3) each adjective-noun combination only appeared once in each list. The order of items within each list was randomized.

All adjective-noun combinations were presented on cards. To help children remember which adjective to use, cards included an emblem at the top (e.g., three coloured circles or a thin and a fat stick figure). In order to familiarize children with the emblems, we first showed them cards with the emblems only, checking whether they knew the target adjective and if not, informing them which words we expected them to use when they saw each emblem.

Children completed the priming task twice, once with French primes and Dutch targets (i.e., French-to-Dutch between-language priming), and once with Dutch primes and French targets (i.e., Dutch-to-French between-language priming). Hence, they encountered the same prime-target pairs in both languages, although in different order (different lists). Each task consisted of three blocks: a baseline and two priming blocks. The baseline was an elicited production task in which children had to describe pictures using adjective-noun orders in the target language.

In the French-to-Dutch task, the baseline was followed by a postnominal priming block where the tester always used the N-Adj order, and then a prenominal priming block, where the Adj-N order was always used. Similar to Experiment 1, the priming blocks of Experiment 2 were set up as a Snap game and children received the same instructions. Children could produce prenominal or postnominal Adj-N combinations in Dutch. The postnominal primes were presented first because these would result in ungrammatical orders in Dutch, the focus of this paper. We feared that presenting the prenominal primes first would result in perseverance effects, preventing any priming of the ungrammatical postnominal order in a subsequent block.

In the Dutch-to-French task, the baseline was also followed by two priming blocks, whereby the French translational equivalent of the adjective used in the Dutch prime was postnominal in the first block and prenominal in the second block. We refer to these blocks as prenominal and postnominal, as in the French-to-Dutch task, for ease of exposition, even though the prime always contained a grammatical prenominal adjective in Dutch. The child had to produce prenominal or postnominal Adj-N combinations in French.

#### Procedure.

Children were tested in two separate sessions, with an interval of approximately one week. Testers were native speakers of Dutch with near native competence in French. In the first session, the tester and the child always spoke in French, except during the priming task, where the child responded in Dutch. The order of tasks was: (1) French vocabulary task; (2) baseline and postnominal block of the French-to-Dutch priming task; (3) French sentence repetition task (as part of a separate project); (4) prenominal block of the French-to-Dutch priming task; and (5) digit span task. In the second session, the tester and child always spoke in Dutch, except during the priming task, when the child responded in French. The order of tasks was: (1) Dutch vocabulary task; (2) baseline and postnominal block of the Dutch-to-French priming task; (3) Dutch sentence repetition task (as part of a separate project); and (4) prenominal block of the Dutch-to-French priming task. This design was intended to increase the activation of the prime language and thus the likelihood of any priming effect. The procedure was otherwise as in Experiment 1. Each session lasted approximately one hour.

#### Coding and Analyses.

Three children participated in the French-to-Dutch session only. In the baseline task in this session six children were unable to independently produce attributive adjective-noun combinations. Their data was therefore excluded from subsequent analyses. This was also the case for two children in the Dutch-to-French task. Our analyses included French-to-Dutch priming data from 24 children and Dutch-to-French priming data from 25 children.

Answers on the priming tasks were coded as *postnominal* (e.g., *dokter rood*, *médicin rouge*, “doctor red”) or *prenominal*. All other responses were coded as *other* (∼3.0% (*N* = 43) in the French-to-Dutch task, and <2.0% (*N* = 29) in the Dutch-to-French task; see S2 in the supplementary materials for a breakdown of the *other* responses). Utterances containing a different adjective than the one targeted were not excluded (e.g., *joli*, “pretty” instead of *heureux*, “happy”). Rather, we coded the French default position for these adjectives (i.e. prenominal or postnominal), based on the literature (e.g., Nicoladis, 2006) and by consulting a university teacher of French (see S3 in the supplementary materials for an overview of children producing a different adjective type than elicited by block and by session).

We analysed the results for the two priming tasks separately. We first built a model with significant fixed effects of *age*, *forward*, and *backward digit span* and random intercepts by *child* and *item*. Fixed effects of *trial number* were excluded from the models, because *trial number* was confounded with *block* (model correlation > .9).

In a second step, we added fixed effects of *block* and *adjective type* as well as their interaction. For *adjective type*, adjectives which appear prenominally in French (1/2) were contrasted with adjectives which appear postnominally in French (reference level, coded as −1/2). For *block*, there were two contrasts: baseline was first contrasted with the postnominal priming block (baseline = 2/3, postnominal block = −1/3, prenominal block = −1/3), and the prenominal priming block was subsequently contrasted with the postnominal priming block (contrast 2: baseline = −1/3, postnominal block = −1/3, prenominal block = 2/3).^5^

Finally, we added fixed effects of children’s Dutch and French vocabulary scores and their interactions with block and adjective type. Because vocabulary scores in the two languages correlated strongly, we tested their effects in separate models.

The statistical analyses were otherwise the same as for Experiment 1.

### Results

#### French-to-Dutch Priming Task.

Table 4 shows the proportion of postnominal N-Adj orders children produced out of the total number of pre- and postnominal adjectives produced.^6^

**Table 4.** Experiment 2, French-Dutch bilingual children: Proportion of Dutch postnominal N-Adj orders children produced in the French-to-Dutch session by French adjective type with standard deviations (in brackets).

		**Block**		
		**Baseline**	**Postnominal**	**Prenominal**
**Adjective type (in French)**	Postnominal	.12 (.29)	.22 (.33)	.11 (.27)
	Prenominal	.10 (.26)	.16 (.34)	.06 (.20)

In a first model including data from all children and the interaction between block and adjective type, the postnominal-baseline block comparison (*B* = −2.00; *SE* = 0.53; *z* = −3.78; *p* < .001) and the postnominal-prenominal block comparison (*B* = −3.01; *SE* = 0.46; *z* = −6.60; *p* < .001) were significant. Children were more likely to produce a postnominal Dutch noun-adjective order in the postnominal priming block than in the baseline and the prenominal priming block. Children were also more likely to produce postnominal noun-adjective orders with Dutch adjectives whose French translation equivalents are postnominal than whose French translation equivalents are prenominal (*B* = −1.23; *SE* = 0.41; *z* = −3.02; *p* < .002). The interactions between block and adjective type were not significant (postnominal-baseline and adjective type: *B* = 0.67; *SE* = 0.98; *z* = 0.68; *p* = .495; postnominal-prenominal and adjective type: *B* = −0.64; *SE* = 0.74; *z* = −0.86; *p* = .388), suggesting that priming in the postnominal block was not affected by the canonical position of the French translation equivalent of the Dutch adjectives produced.

Influence analyses identified four children with Cook’s distance scores that stood out (i.e., higher than 4 divided by the 24 children in the sample). Closer inspection showed that with the exception of a single response by one other child, these children were the only ones who produced postnominal noun-adjective orders in the baseline and the prenominal priming block. Further inspection of the French baseline data (session 2) for these children showed that they also used prenominal adjectives in postnominal position most of the time, suggesting that they had not yet acquired the correct placement for the French prenominal adjectives studied here.

**Figure 3.**
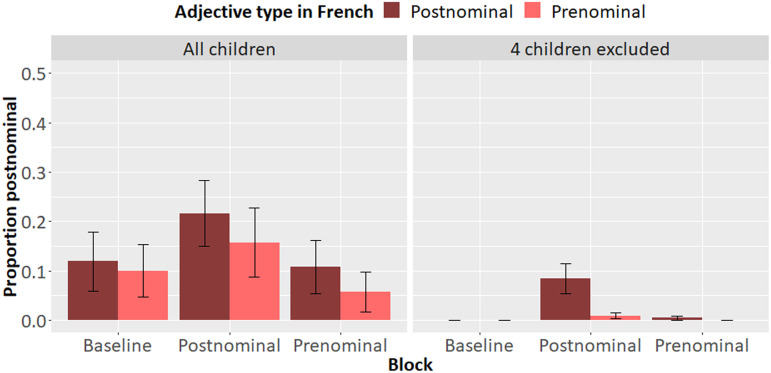
Experiment 2, French-Dutch bilingual children: Proportion of Dutch postnominal N-Adj orders children produced in the French-to-Dutch session by French adjective type including all children (left panel) and excluding influential cases (right panel).

Figure 3 presents the results for all children (left panel) and without influential cases (right panel). Nine of the remaining 20 children whose data is shown in the right panel produced 20 postnominal noun-adjective word orders in the postnominal priming block, of which 18 were with adjectives in Dutch whose translation equivalent in French was postnominal. The model for the postnominal priming block only, excluding the influential cases (Table 5) revealed a significant effect of adjective type, suggesting that children who do not normally produce ungrammatical postnominal N-Adj orders in Dutch—at least, as measured by our baseline—can be primed to do so. Crucially, such orders virtually always occurred with Dutch adjectives whose French translation equivalent appears in postnominal position. Furthermore, the older children were, the less likely they were to produce postnominal N-Adj orders.

**Table 5.** Experiment 2, French-Dutch bilingual children: Summary generalized linear mixed effects model of children’s proportion of postnominal orders in the French-to-Dutch priming session for the postnominal priming phase excluding influential cases.

**Random effects**				
**Groups**	**Name**	**Variance**	***SD***	
Participant	(Intercept)	1.055	1.027	
Items	(Intercept)	3.251	1.803	
Number of observations: 461	Participants: 20			
**Fixed effects**				
	***B***	***SE***	***z***	***p***
(Intercept)	−5.78	1.20	−4.83	< .001
Adjective type (French)	−2.49	1.10	−2.26	.024
Age	−0.95	0.33	−2.90	.004

#### Vocabulary.

Separate analyses with children’s Dutch and French vocabulary scores and including all children showed that vocabulary scores interacted significantly with the first block contrast (Dutch vocabulary: *B* = 0.09; *SE* = 0.03; *z* = 2.73; *p* = .006; French vocabulary: *B* = 0.10; *SE* = 0.04; *z* = 2.86; *p* = .004) and marginally significantly with the second block contrast (Dutch vocabulary: *B* = 0.05; *SE* = 0.03; *z* = 1.91; *p* = .056; French vocabulary: *B* = 0.06; *SE* = 0.03; *z* = 1.68; *p* = .093). The less proficient children were in Dutch and French, the stronger they were primed with the postnominal French word order. Overall patterns remained the same after excluding the four children who produced postnominals in the baseline.

#### Dutch-to-French Priming Task.

Table 6 shows the proportion of prenominal Adj-N orders children produced in French out of the total number of pre- and postnominal adjectives produced. Overall, children produced more prenominal orders with prenominal adjectives than with postnominal adjectives. This was confirmed in the analysis (Table 7): The fixed effect of adjective type was significant. There were no observable differences between blocks. This was also confirmed in the analyses: neither the first nor the second block contrast, nor the interactions between block and adjective type approached significance. In other words, there was no significant priming effect.

**Table 6.** Proportion of French prenominal Adj-N orders children produced in the Dutch-to-French session by French adjective type with standard deviations between brackets.

		**Block**		
		**Baseline**	**Postnominal**	**Prenominal**
**Adjective type (in French)**	Postnominal	.43 (.42)	.39 (.39)	.40 (.39)
	Prenominal	.65 (.39)	.61 (.41)	.62 (.39)

**Table 7.** Summary generalized linear mixed effects model of children’s proportion of prenominal orders in the Dutch-to-French priming session (data from three influential children removed).

**Random effects**				
**Groups**	**Name**	**Variance**	***SD***	
Participant	(Intercept)	10.393	3.224	
Items	(Intercept)	0.248	0.498	
Number of observations: 1293	Participants: 22			
**Fixed effects**				
	***B***	***SE***	***z***	***p***
(Intercept)	0.33	0.70	0.47	.635
Baseline vs. postnominal block (contrast 1)	0.37	0.29	1.26	.207
Prenominal vs. postnominal block (contrast 2)	0.06	0.21	0.29	.776
Adjective type (French)	2.04	0.25	8.30	< .001
Contrast 1: Adjective type	0.09	0.58	0.15	.877
Contrast 2: Adjective type	0.01	0.42	0.03	.974

#### Vocabulary.

Separate analyses with children’s Dutch and French vocabulary scores showed that children’s vocabulary scores in both languages were significantly related to the first block contrast (Dutch vocabulary: *B* = −0.05; *SE* = 0.02; *z* = −3.00; *p* = .003; French vocabulary: *B* = −0.02; *SE* = 0.01; *z* = −2.3; *p* = .021). With lower vocabulary scores, the difference between the baseline and the postnominal priming block became larger. However, after removing three influential children, the interactions became weaker or even changed direction and no longer approached significance (Dutch vocabulary: *B* = 0.02; *SE* = 0.02; *z* = 0.8; *p* = .448; French vocabulary: *B* = −0.01; *SE* = 0.01; *z* = −0.61; *p* = .545).

The interactions between children’s vocabulary scores and adjective type were significant (model without 3 influential children: Dutch vocabulary: *B* = 0.05; *SE* = 0.02; *z* = 2.75; *p* = .006; French vocabulary: *B* = 0.04; *SE* = 0.01; *z* = 3.98; *p* < .001). With better vocabulary the difference between adjective types—more prenominal word orders with prenominal adjectives and more postnominal word orders with postnominal adjectives—became larger. Neither the fixed effect of vocabulary score, nor any of the other interactions with vocabulary approached significance.

### Discussion

The results of the French-to-Dutch session in Experiment 2 mirror the results of Experiment 1. First, we observed priming of the postnominal N-Adj word order from French into Dutch. This is in line with our hypothesis based on Hsin et al. (2013), as well as previous studies with bilingual adults showing that word order overlap between languages is not required for between-language priming to take place (e.g., Chen et al., 2013; Shin & Christianson, 2009). It is furthermore consistent with the proposal that cross-linguistic influence results from between-language priming (e.g., Serratrice, 2016). Second, we found that priming effects were stronger, the less proficient children were in their two languages, again in line with our hypothesis. Moreover, the observation that it was the children with lower proficiency in their two languages who were more susceptible to priming supports findings from other studies showing larger priming effects in less skilled speakers, including both monolingual children and adult second language learners (Hartsuiker & Kolk, 1998; Leonard et al., 2000; Rowland et al., 2012).

Importantly, we found that between-language priming of ungrammatical adjective placement was lexically restricted. First, children produced more ungrammatical postnominal orders in Dutch with adjectives that are typically postnominal in French (e.g., *groen*, “green”) than with adjectives that are typically prenominal (e.g., *groot*, “big”), irrespective of the structure they were primed with. Nicoladis (2006) observed a similar (marginally significant; *p* = .08) effect in English as well for French-English bilingual children. Second, and crucially, once we removed the data from four influential children who produced ungrammatical postnominal N-Adj orders in Dutch in the baseline, the priming effect for the remaining children was *only* visible for Dutch adjectives whose French translation equivalent is postnominal. In line with our findings, the model put forward by Nicoladis (2006) would predict production and priming of postnominal N-Adj orders in Dutch only with Dutch adjectives whose French translation-equivalent is postnominal. In other words, the results of Experiment 2 suggest that priming of ungrammatical adjective placement in Dutch happens indirectly via the co-activation of postnominal adjectives in French.

The results of the Dutch-to-French session were not in line with any of our hypotheses. We found no evidence of structural priming. When children produced incorrect French prenominal Adj-N orders, this was irrespective of whether a prenominal Dutch prime was present. Simply put, we found evidence for cross-linguistic influence from Dutch to French resulting in ungrammatical structures, in line with previous studies using other methods (e.g., Granfeldt, 2000; Nicoladis, 2006; Prévost, 2009), but we found no evidence of priming of these structures. These findings contrast with our results in Experiment 1 and in the French-to-Dutch session, as well as with those of Hsin et al. (2013). We explain the absence of any priming effects as follows: First, children incorrectly produced French postnominal adjectives in prenominal position in the baseline at a rate which was considerably higher (43%) than the 3-to-5-year-old French monolingual children (2%) in Nicoladis (2006), suggesting that many of them were influenced by their knowledge of Dutch. In other words, because of frequent exposure to Dutch prenominal structures over time, there was cross-linguistic influence in the baseline already. Second, the absence of any ‘direct’ priming effects was most probably a ceiling effect. Many of the children who might have been expected to show the greatest priming effects (i.e., with low proficiency and therefore less stable representations) already placed postnominal adjectives in the baseline in prenominal position. For these children, then, there was simply no room left for priming.

The bilingual French-Dutch children in our study were not primed to use the ungrammatical Adj-N order with postnominal adjectives in French. This contrasts with the bilingual Spanish-English children in Hsin et al., who were primed to produce this order in Spanish. The discrepancy between these two sets of findings could be due to the prenominal Adj-N order being more frequent in French than it is in Spanish (e.g., Rizzi et al., 2013), where it is possible but only in restricted contexts (see fn. 1). As such, the Spanish-English bilingual children in Hsin et al. (2013) might have produced more correct postnominal orders in Spanish in the absence of any priming than the French-Dutch children in French—due to the Spanish N-Adj order being less vulnerable to cross-linguistic influence from English because of its higher occurrence than the French N-Adj order. If this is correct, there would have been more room left for priming in the children in Hsin et al. (2013) compared to the children in our study.

## GENERAL DISCUSSION

This study investigated the extent to which bilingual children could be primed to use ungrammatical structures in one of their languages under influence from the other, whether such priming is lexically restricted, and to what extent language proficiency affected the strength of priming.

### Priming of Ungrammatical Structures

Despite a number of methodological differences between Experiment 1 and Experiment 2 in number, type and presentation of items, lexical overlap, and proficiency tasks, we observed consistent findings across both experiments. As we hypothesized, the experiments showed that the ungrammatical postnominal N-Adj order can be primed from French and Spanish into Dutch. Furthermore, in our second experiment we found evidence that priming of Dutch adjective orders depended on the canonical position of adjectives’ translation equivalents in French. The observed priming effects align well with the proposal that cross-linguistic influence is due to long-term effects of between-language priming (e.g., Serratrice, 2016; see also van Gompel & Arai, 2018). Importantly, together with Hsin et al. (2013), we show that this claim is not limited to grammatical structures (Unsworth, in press; Vasilyeva et al., 2010; Wolleb et al., 2018). More specifically, these results suggest that frequent exposure to a structure in Language A may prime the structure for use in Language Alpha, even if said structure is not available in Language Alpha. Such long-term between-language priming may lead to qualitative differences in language use between bilingual and monolingual children (e.g., Strik & Pérez-Leroux, 2011) and has more generally been proposed as a driving force in contact-induced language change (Fernández et al., 2017).

How best to account for the priming of ungrammatical structures? The finding that between-language priming of ungrammatical structures was lexically restricted aligns well with the account put forward by Nicoladis (2006, 2012, Nicoladis et al., 2010; cf. the ‘broad sharing’ approach proposed by Hsin et al., 2013). On this account, ungrammatical structures result from the co-activation of separate representations indirectly connected via lemmas in each language through shared conceptual representations (cf. Figure 2). In other words, the production of Dutch adjectives that have a French postnominal translation equivalent (e.g., *groen-vert*, “green”) activates the French postnominal N-Adj representation connected to the French adjective through the shared conceptual representation for *green*. Consequently, this (ungrammatical) structure competes for selection with the (grammatical) Dutch Adj-N representation directly connected to the Dutch lemma. Strikingly, priming of ungrammatical constructions (within a language) has also been found to be lexically constrained by Ivanova and colleagues (Ivanova et al., 2012). We leave the question of whether these two sets of findings reflect the same effect to future research.

**Figure 4.**
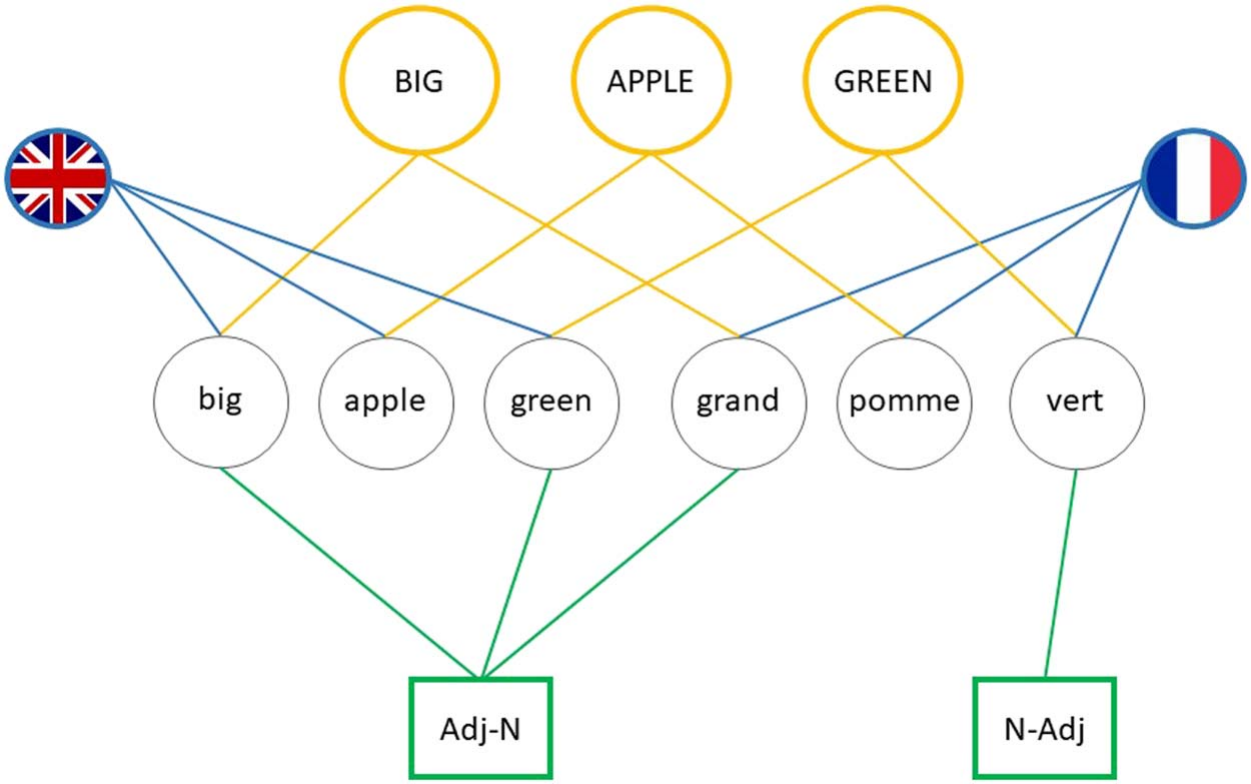
Schematic representation of the English adjective lemmas *big* and *green* and the French adjectives *grand* and *vert* and their connections to their conceptual, structural and language nodes. Adapted from Hartsuiker et al. (2004).

Whilst the approach put forward by Nicoladis neatly accounts for the lexically restricted nature of the observed priming effect, it is not entirely unproblematic. We see (at least) two issues with this proposal in its present form. First, it considers instances of cross-linguistic influence as speech *production* errors. Given that both cross-linguistic influence and between-language priming occur in comprehension (e.g., Bosch & Unsworth, 2021; Nitschke et al., 2014), such a view seems too restrictive. Hence, Nicoladis’ account needs extending to sentence comprehension. Second, her proposal does not specify how exactly an ungrammatical structure (syntactic ‘frame’ or combinatorial node, depending on the model) is selected. We believe the mechanisms proposed to account for grammatical priming in the shared-syntax model (Hartsuiker et al., 2004) could in fact also account for priming of ungrammatical structures. On this residual-coactivation account of priming, the amount of activation a structure receives determines whether or not it is selected over another structure. Activation depends on the strength of the connections between lemmas and structural nodes (e.g., Kootstra & Doedens, 2016) and whether the structure has recently been selected (i.e., has been primed). If we apply this logic to Nicoladis’ account, an N-Adj node becomes activated when a lemma is selected that has a connection to this node (e.g., French *vert* or its Dutch translation equivalent *groen*). Furthermore, activation of the N-Adj node is strongest if the N-Adj node has been recently selected (i.e., primed) due to residual activation left on the node. As a result, the N-Adj node potentially receives more activation than the Adj-N node and could be selected even when ungrammatical. We schematically show this in Figure 4, augmenting the shared-syntax model in Figure 1 that contains shared structures only (Hartsuiker & Bernolet, 2017; Hartsuiker et al., 2004) with a language-specific structural node (N-Adj).

In Figure 4, priming of (grammatical) prenominal Adj-N orders from English to French is predicted to occur through the activation of the shared Adj-N node between English adjectives and French prenominal adjectives (e.g., *big-grand*), in line with shared syntax (e.g., Hartsuiker et al., 2004). In contrast, the postnominal N-Adj order is only directly connected to French postnominal, but not to French prenominal and English adjectives, in line with Nicoladis (2006). There are two differences between Figure 4 and Nicoladis’ (2006) account of ungrammatical cross-linguistic influence that are important to mention. First, on Nicoladis’ (2006) account, the Adj-N node is not shared between languages (also see Figure 2). Our results do not directly speak to the question of whether the Adj-N node is shared but given the available evidence that similar word orders become shared between languages (e.g., Hartsuiker et al., 2004; Janciauskas & Chang, 2018), we assume that this is the case. Future research testing the priming of both grammatical and ungrammatical structures in the same participants may shed light on this question. Second, on the shared-syntax account, language nodes are connected to lemmas only, whereas the Adj-N and N-Adj orders in Nicoladis (2006) are also directly connected to language nodes. Whilst the presence or absence of connections between language and structural nodes does not impact our conclusions, it does have consequences for an alternative explanation of ungrammatical priming, namely, priming as code-switching, which we discuss in more detail below.

There are several alternative explanations for why ungrammatical structures can be primed, although we are not aware of any that can directly explain the lexical restrictions we observed. First we consider code-switching. One account of code-switching is the Matrix Language Frame model (Myers-Scotton, 1993). On this account, the matrix language (French in the context of the French-to-Dutch priming in Experiment 2) determines the structure of the utterance and the embedded language (i.e., Dutch) provides the lexical items. When *overtly* code-switching (e.g., *une pomme green*), items are selected from both languages. In our experiment, children may have been *covertly* code-switching, that is, they may have used the structural representation from French, which was more readily available for use due to recent activation, and inserted lexical items from Dutch (e.g., *een appel groen*, “an apple green”). Priming as code-switching could thus be part of the explanation for the priming of ungrammatical structures. It cannot, however, account for the lexical restrictions we observed.

A second alternative explanation for our findings as suggested by an anonymous reviewer is that what may have been primed in our experiment is not so much the postnominal N-Adj order, but a more general postnominal attribute order, which is available in Dutch in the form of a postnominal prepositional phrase (PP) modifying a noun phrase (NP; e.g., de kat van de astronaut, “the cat of the astronaut”). On such an account (e.g., Jacob et al., 2017), what is primed is constituent order (i.e., a right-branching NP: NP-X) rather than the structural N-Adj node. However, aside from the fact that it is unclear how this account can explain lexical restrictions in priming, we do not see how the availability of this order more generally in Dutch should lead to an adjective being inserted in postnominal position given that this particular constituent order is ungrammatical. To be more precise, even if a French postnominal N-Adj order can prime a Dutch postnominal N-PP order—because both are postnominal attributes—it is unclear how the PP in Dutch can be replaced by an adjective. Moreover, under a constituent-priming account, Dutch possessive NP-PP structures which were present in Experiment 1 as fillers should have primed Dutch N-Adj orders in the Dutch-to-Dutch priming session, and this was not the case.

A third alternative explanation for the priming of ungrammatical structures is the “copy-and-edit” strategy put forward by Hartsuiker and Bernolet (2017). This entails that low-proficiency speakers who are uncertain about the target sentence they must produce might explicitly recall the sentence they just heard in the prime and adapt it, by inserting words from the target language. This explanation can account for the negative relation we found between children’s target language proficiency and priming effects, but not for the lexical restrictions we observed.

A final alternative explanation, suggested to us by an anonymous reviewer, is that what is primed is not word order, but word category. This would be a more general task effect whereby a “noun-first” strategy is primed in the postnominal French-to-Dutch priming session: upon hearing the tester use a French noun at the start of their picture description, children might have done the same thing in Dutch, automatically leaving the adjective until last. There is some support for this explanation: the Dutch postnominal adjectives children produced were never marked for gender (e.g., *hond_COMMON_ rood* instead of *hond_COMMON_ rod-e_COMMON_*, “dog red”; cf. fn. 3 and fn. 6). It is therefore difficult to know whether the postnominal responses in Dutch are similar to ‘real’ N-Adj structures in French or whether they are rather ‘structureless’ N-Adj sequences in line with a noun-first strategy. Whilst we cannot rule out the latter option, this, too, seems unlikely given the observed lexical restrictions. Furthermore, even if children were adopting a “noun-first” strategy, they still could have used a grammatical Dutch structure to describe their pictures, namely, by adding a predicative adjective (e.g., *een apple die groen is*, “an apple that is green”). Such utterances occurred occasionally (*n* = 19, produced by two participants) but not as frequently as the N-Adj responses. It seems, therefore, that a noun-first strategy can also not explain children’s production of *ungrammatical* N-Adj orders.

In sum, whilst we cannot rule out these alternative accounts, none can explain the lexical restrictions observed in Experiment 2, unlike the integrated approach given in Figure 4.

### Bilingual Proficiency and Priming

The second aim of this study was to investigate whether priming of ungrammatical structures was related to proficiency, and if so, how. We observed that priming of the postnominal N-Adj structure from Spanish and French into Dutch was stronger with lower proficiency. This finding fits with an error-based learning account of priming of ungrammatical structures: children with lower levels of proficiency in their languages have less stable structural representations, which are more susceptible to change (e.g., Peter et al., 2015; Rowland et al., 2012). Hence, lower proficiency in the *prime* language results in greater surprisal and thus larger priming effects (e.g., Chang et al., 2006). Lower proficiency in the *target* language allows for the selection of a structure in the prime language over a grammatical structure in the target language. Note that the latter effect is caused by a different mechanism than prime surprisal. Rather, it is due to activation strength and selection (e.g., Nicoladis, 2006): the weaker a target structure is represented (i.e., the Dutch prenominal Adj-N order), the weaker its activation, and the more likely a structural representation from another language can become selected instead of the target structure (i.e., the French postnominal N-Adj order), especially when the non-target structure has been primed.

More generally, our study highlights how examining proficiency effects in bilingual children is not quite as simple as for (sequential) bilingual adults, who are almost always late sequential bilinguals and hence have already fully acquired one language. Bilingual children, on the other hand, are developing two languages in parallel, and even when they are first exposed to both languages from birth, the rate of development can vary considerably across the two (Hoff et al., 2012). Consequently, it is essential to include proficiency measures in both languages (cf. sequential bilingual adults, where a measure of L2 proficiency alone is sufficient). In this study, we chose absolute measures of language proficiency, but there may be instances where relative measures combining scores from both languages make more sense.

Finally, our examination of the response patterns of individual children revealed that it is not only children’s general language proficiency which is of importance, but also their knowledge of the target structure(s) in question. Language learners of all ages may pass through developmental stages where they consistently make certain errors, and when it comes to assessing priming behaviour, this can complicate matters considerably. For a comprehensive evaluation of the relation between proficiency, priming and knowledge of specific syntactic structures, longitudinal data are needed.

In conclusion, this study has drawn on insights from two fields which by and large remain isolated from each other, despite being concerned with many similar questions. We examined one of the perennial and as yet unanswered questions in the field of child bilingualism through the lens of adult bilingual priming. Doing so enabled us to gain a clearer understanding of the mechanisms underpinning cross-linguistic influence in bilinguals—both children and adults—although there is admittedly still plenty of detail to be fleshed out if we are to arrive at a comprehensive picture of the relation between cross-linguistic influence, between-language priming and bilingual proficiency. A careful examination of individual children’s behaviour led to a better grasp of the complex interplay between proficiency and priming as it relates to the production of ungrammatical structures by bilinguals. We hope that our results will encourage researchers working with bilingual adults to pay more attention to individual differences in future priming studies.

## ACKNOWLEDGMENTS

We would like to thank all participating families for their time, student assistants for data collection, Dr. Janine Berns for her input on the French adjectives, Elise van Wonderen for her invaluable help in better understanding contrast matrices, and to audiences at COM and ISBPAC for their constructive feedback on an earlier version of this paper.

## AUTHOR CONTRIBUTIONS

Chantal van Dijk: Study conception and design, supervising data collection, statistical analyses, writing and revising manuscript. Sharon Unsworth: Study conception and design, supervising data collection, writing and revising manuscript.

## DATA AVAILABILITY STATEMENT

All data and code can be found at https://osf.io/nk93f/?view_only=d1be4afb7ad1457ea5184dc63d8f3da3.

## FUNDING INFORMATION

This work was supported by the Dutch Research Council [grant number 23000407, awarded to Sharon Unsworth.

## Notes

^1^ Adjectives in Romance languages can be placed in prenominal *and* postnominal position. Typically, the postnominal position yields a literal meaning of the adjective, whereas the prenominal position generally yields a figurative or more poetic meaning (e.g., Demonte, 2008). We did not test this semantic distinction and our participants were not old enough to have (fully) acquired it (see Rizzi et al., 2013). We therefore refer to any mismatch between a literal meaning (e.g., an apple with a green colour) and its default position as ungrammatical.^2^ Random slopes and correlations between random slopes resulted in convergence errors and were therefore removed.^3^ Note that none of the postnominal adjectives agreed in gender with their noun. In Dutch, attributive adjectives modifying a common noun show agreement marked with a schwa (e.g., *hond_COMMON_ rod-e_COMMON_*, “dog red”). However, even though all target nouns in this experiment were common, none of the postnominal adjectives showed such gender agreement (e.g., *hond_COMMON_ rood*, “dog red”). In contrast, almost all prenominal adjectives *were* marked for gender.^4^ Initially, we used *mince*/*dun* ‘thin’ instead as a prenominal adjective. However, after learning that this adjective typically appears postnominally we replaced it with *beau*/*mooi* ‘beautiful’.^5^ The contrasts specified here do not directly reflect our hypothesis matrix, namely that we first compare the postnominal priming block to the baseline (baseline = −1, postnominal block = 1, and prenominal block = 0) and then the postnominal block to the prenominal block (baseline = 0, postnominal block = 1, and prenominal block = −1). Instead, our contrasts are non-orthogonal (correlated). As a consequence, the simple contrasts used in this study are the generalized inverse of the hypothesis matrix. We refer the reader to Schad et al. (2020) for an elaborated discussion of how to convert the hypothesis matrix to the contrast matrix.^6^ Note that similar to in Experiment 1, none of the Dutch postnominal adjectives were inflected for gender (e.g., *hond_COMMON_ rood*) but almost all prenominal adjectives were.
