# Supplementary material for: On the Relation Between Cross-Linguistic Influence, Between-Language Priming and Language Proficiency: Priming of Ungrammatical Adjective Placement in Bilingual Spanish-Dutch and French-Dutch Children
Source: Open Mind (Camb). 2023 Sep 20;7:732–56. doi: 10.1162/opmi_a_00105 (PMC10575554; doi:10.1162/opmi_a_00105)
Supplement: Supplementary file 1 [file opmi-07-732-s001.docx]

**Supplementary materials**

**S1 – “other” responses in Experiment 1**

There were 30 responses in Experiment 1 that were classified as “other”. The 30 ‘other’ items were:

- a relative clause (e.g., *de hond die rood is*, “the dog that is red”) (*n* = 19, produced by two participants);
- type *de hond in het rood* (“the dog in red”) (*n* = 6, all produced by the same participant);
- type *de hond die rode* (“the dog, the red one”) (*n* = 3, all produced by the same participant, who otherwise only produced relative clauses)
- other (*n* = 2)

Each of these (except the ‘other’) could potentially be seen as word order priming.

**S2 – “other” responses in Experiment 2**

The responses that were marked as “other” in Experiment 2 (in the dataset that was analysed) were:

**Session 1 – French-to-Dutch:**

- Baseline: *n* = 0
- Postnominal priming block: *n* = 12
  - Predicative use (e.g., *kip die rood is*, “chicken that is red”): *n* = 12 (10 produced by 1 participant, other two by 2 participants)
- Prenominal priming block: *n* = 12
  - Predicative use: *n* = 5 (4 produced by same 1 child, 1 by other child)
  - Type *kip die huilt* (“chicken that cries”): *n* = 3
  - Type *baby-aapje* (“baby-monkey”): *n* = 4

**Session 2 – Dutch-to-French:**

- Baseline: *n* = 0
- Postnominal priming block: *n* = 6
  - Predicative use: *n* = 1
  - Other (not relevant): *n* = 5
- Prenominal priming block: *n* = 3
  - Other (not relevant): *n* = 3

**S3 – Responses with a different adjectives type than elicited in Experiment 2**

This table gives an overview of how often children produced a different adjective type than the type of adjective we elicited (e.g., prenominal *grand* instead of postnominal *vert*):

|  | **Elicited adjective type** | **Adjective type used by child** | **Count** |
| --- | --- | --- | --- |
| **French to Dutch session** | postnominal | prenominal | 3 |
|  | prenominal | postnominal | 0 |
| **Dutch to French session** | postnominal | prenominal | 27 |
|  | prenominal | postnominal | 6 |

This did not happen very often, with the exception of the production of a prenominal adjective instead of a postnominal adjective in the Dutch-to-French session (27 times out of 857 prenominal adjectives that we elicited). When we break these numbers down by block and adjective-noun order the children produced, we see the following:

|  | **Order produced** | **Count** |
| --- | --- | --- |
| **Baseline** | postnominal | 4 |
|  | prenominal | 1 |
| **Postnominal priming block** | postnominal | 4 |
|  | prenominal | 2 |
| **Prenominal priming block** | postnominal | 6 |
|  | prenominal | 9 |

As these numbers show, there is not a clear pattern. Children did not seem to have changed a French postnominal adjective into a French prenominal adjective in order to create a correct prenominal French Adj-N order to match the Dutch prenominal prime.
